# A New Degree

**Published:** 1894-02

**Authors:** 


### EDITORIAL.

### A New Degree.

At a recent meeting of the Regents of the University of Michigan, at the suggestion of the Faculty of the Dental Department, the degree of Doctor of Dental Science was established, to be conferred upon those who after completing the regular three years' course in a satisfactory manner and graduating, receiving the degree of Doctor of Dental Surgery, take another year's work, which will embrace advance work and original work as well on certain prescribed lines. It will be required in order to do this that the applicant shall have made well-nigh perfect work in his regular course. The course indicated for this degree in the scheme presented is work in materia medica, embracing laboratory work in organic chemistry, laboratory work in physiology, original work on these lines, and with special reference to some dental remedy; also in pathology, embracing embryology and histology, bacteriology, laboratory course and original research on dental diseases.

This course would add very greatly to the equipment of the dental practitioner; it would give him attainments and ability much in advance of the general graduates in dental surgery. These who have given attention to the ordinary practitioners of dentistry as they go out from the usual course of instruction, cannot avoid being impressed with the great want that is so frequently experienced by the beginner when he is confronted by many of the more severe and obscure cases of diseases of the teeth and mouth.

The object in establishing this course is, if possible, to increase the ability of the beginner in dental practice. That something more is required in this respect than is obtained, not only in dental but in medical colleges as well, in the ordinary course, is fully demonstrated by the establishment of Post Graduate Departments, especially in medicine in nearly all our larger cities, and there seems to be a call for such schools in dentistry. The efforts as yet, however, have been quite circumscribed, having reference only to some particular line of instruction, as for example: Prosthetic Dentistry, or the more practical departments of work.

